# Coloured random graphs explain the structure and dynamics of cross-linked polymer networks

**DOI:** 10.1038/s41598-020-71417-9

**Published:** 2020-09-03

**Authors:** Verena Schamboeck, Piet D. Iedema, Ivan Kryven

**Affiliations:** 1 Van ’t Hoff Institute for Molecular Sciences, University of Amsterdam, Science Park 904, 1098 XH Amsterdam, The Netherlands; 2 Mathematical Institute, Utrecht University, PO Box 80010, 3508 TA Utrecht, The Netherlands; 3 Centre for Complex Systems Studies, 3584 CE Utrecht, The Netherlands

**Keywords:** Chemical engineering, Complex networks, Phase transitions and critical phenomena, Statistical physics

## Abstract

Step-growth and chain-growth are two major families of chemical reactions that result in polymer networks with drastically different physical properties, often referred to as hyper-branched and cross-linked networks. In contrast to step-growth polymerisation, chain-growth forms networks that are *history-dependent*. Such networks are defined not just by the degree distribution, but also by their entire formation history, which entails a modelling and conceptual challenges. We show that the structure of chain-growth polymer networks corresponds to an edge-coloured random graph with a defined multivariate degree distribution, where the colour labels represent the formation times of chemical bonds. The theory quantifies and explains the gelation in free-radical polymerisation of cross-linked polymers and predicts conditions when history dependance has the most significant effect on the global properties of a polymer network. As such, the edge colouring is identified as the key driver behind the difference in the physical properties of step-growth and chain-growth networks. We expect that this findings will stimulate usage of network science tools for discovery and design of cross-linked polymers.

## Introduction

Already in the early days of network science it has been realised that in dynamic networks the entire time trajectory of network formation may reflect on the topological features of the structure that is formed at the end. In other words, that the structure of a dynamic network may have memory of its past. Some examples of evolving network models include Price’s (1965) preferential attachment model^1^, the vertex copying model^2^, network optimisation models^3^, and branching simlicial complexes^4,5^. Preferential attachment, vertex copying models, and branching simplicial complexes all result in heavy-tailed degree distributions. In this work, we introduce an evolving network model with an arbitrary degree distribution to show that the different effects induced by the two most common polymerisation processes on the resulting materials are provoked by the presence or absence of memory in the underlaying network structures.

*Step-growth* and *chain-growth* polymerisation are two major families of chemical reactions that result in polymer networks. For step-growth, such polymers include polyethylene (PE), polyvinyl chloride (PVC), polypropylene (PP) and polyacrylates^6^, which have a variety of applications: including paints and adhesives^7,8^, food packaging^9^, biomaterials and medical devices^10,11^. Well-known examples of *chain-growth* polymers are gels that find applications as absorbents for medical, chemical and agricultural purposes^12^, and coatings made by photopolymerisation^13,14^. The reason for the difference in the physical and mechanical properties of the step- and chain-growth derived polymers lies in the distinct network structures. Moreover, the structure of chain-growth networks can be manipulated by adjusting polymerisation conditions and species concentrators in order to optimise physical and mechanical properties.

The structure of networks that are produced by step-growth polymerisation is well understood. The first models were developed by Flory^15^ and Stockmayer^16^ in the 1940s. These models represent a monomer unit as a vertex with a defined number of half-edges, and rely on the assumption that any pair of half-edges has independent and identical probability to be connected (the i.i.d. assumption). Such models predict molecular size distributions and the gelation time for several special cases of monomer functionality. Following the work by Flory and Stockmayer, branching and coagulation processes were used to derive the size of gel molecules and predict the time when the gel starts to form, see Gordon^17,18^ and Ziff^19,20^. In our recent works^21–24^, we continued this avenue by identifying the entire polymer network as a random graph with a given degree distribution^25^, which provides a new theory and algorithms of general step-growth polymer systems irrespective of the number of monomers and their functionality. However successful such a technique proves to be for step-growth polymer networks, it cannot explain the networks produced by chain-growth procedure due to history dependence of the latter.

In the polymer context, the idea of *history-dependence*^26^ means that network structures formed by chain-growth polymerisation violate the i.i.d. assumption and thus such networks have a different structure from that of step-growth polymers. Due to the history-dependence of the network structure, chain-growth polymerisation is typically modelled with master equations, also known as population balance equations (PBEs), which track the evolution of several network properties throughout the entire formation process^27–30^. Stochastic simulation algorithms (SSAs)^31^ provide another alternative, which is especially suitable for complex polymerisation systems with many functional groups^32^. The downside of SSA is that it may require prohibitive computational costs, especially when important species, such as radicals, are present in small relative concentrations.

One striking feature of history-dependent processes is that the moment in time when an extensive cluster emerges, that is the gel point^13^, differs from the percolation threshold found when randomly removing bonds in the final network. This provides a strong contrast to network formation by step-growth polymerisation, where the polymerisation process is equivalent to random percolation on the final network topology^21,23,24^. In some cases, however, the uncoloured random graph gives a surprisingly good estimate of the phase transition and the gel fraction^13^, which suggests that under certain conditions chain growth polymerisation may not induce a significant history dependency. Beyond polymers, strong history dependence is characteristic to processes on networks that can be reformulated as an annealed dynamics^33^.

In “The coloured directed random graph with arbitrary degree distribution” section the main results of the theory for coloured directed random graphs are summarised. We illustrate the concept on two types of chain-growth polymerisation, living polymerisation and free-radical polymerisation. We report a procedure to compute molecular weights of the sol polymer and the gel fractions, and compare these quantities with stochastic simulations. “Example: living polymerisation” section presents the results for linear and nonlinear living polymerisation, and in “Example: free-radical photopolymerisation” section the methodology is applied to linear and non-linear free-radical polymerisation. “Conclusion” section discusses the conclusions of the present work. In “Methods ” section, the derivation of the weight-average molecular weight and the gel fraction in the coloured directed random graph is provided, as well as derivations of the kinetic schemes and master equations for the living and free-radical (photo-) polymerisations.

## Results

In the present work we represent polymers as graphs. The nodes represent the monomer units and the edges correspond to the covalent bonds, which are formed during the polymerisation process. The degree distribution *u*(*k*) is the probability of a uniformly at random selected node to have *k* incident edges. Many macroscopically observable quantities in polymer chemistry have their equivalents in the graph theory: the gel point corresponds to the phase transition at which the giant component emerges; the gel fraction is the relative size of the giant component, and the molecular weight is the expected size of (weakly) connected components.

Many real world networks evolve due to a local temporal process—a set of rules that connects nodes depending on their labels, degrees, etc. Such a local formation process may or may not result in a structure that is significantly different from a random graph with i.i.d. degrees. Step-growth polymerisation is one example of a network formation process that only constrains the degree distribution, beyond that, the graph is fully random and history-independent. Such networks can be model by a random graph that satisfies a fixed degree distribution at each time point^21,23,24^. However, if the emerging structure depends on history of its formation, it cannot be represented by degree distribution alone, as shown in the following example: Consider a system with linear polymer chains having a narrow length distributions. The degree distribution only contains information about the number of nodes with degree two, and the nodes with degree one. If nodes with fixed degrees one and two are connected randomly, we will find chains with many different lengths, therefore overestimating the variance of the length distribution.

We extend the ‘amount of information’ stored in the degree distribution by representing the time intervals at which each edge was created with edge colours. We discretise time into defined time intervals $\Delta t_i=[t_{i-1},t_i), \, i=1,\dots ,N$ with every time interval $\Delta t_i$ being attributed colour *i*. As before, all pairs of half-edges are assigned an equal probability to connect, but now only half-edges, which have the same colour, are paired. In the limit of infinitely small time intervals, every edge is coloured in a different colour, which would allow exact reconstruction of the polymer structure. Hence, we propose that extending the degree distribution to a coloured degree distribution, should allow us to apply random graph modelling to history-dependent networks.

**Figure 1:**
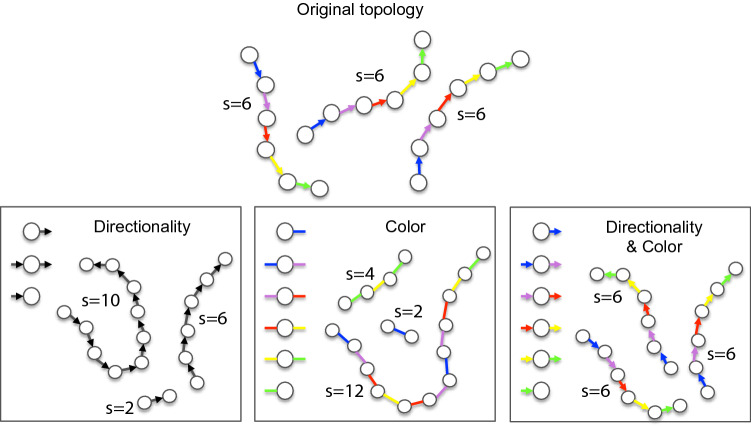
Representing bond formation time as edge colour in directed random graphs. *Top*: An example of a coloured directed graph. *Bottom*: Reconstructed possible structures with: uncoloured directed random graph (*left*), the coloured undirected random graph (*middle*), and the coloured directed random graph (*right*).

In Fig. 1, we illustrate the concept on a simple example of linear polymer chains: The original structure that we aim to recover from the random graph consists of three polymer chains of length 6. We take degree distributions of different random graph models: (1) directed, (2) undirected and coloured, (3) directed and coloured. If the degree distribution only contains information on the number of half-edges (degree) and their orientation (*left*), chains of various lengths are obtained. In the second case, the degree distribution contains information on the degree and the colour, but the edges are considered undirected (*middle*), again, various chain lengths are obtained. Only if the degree distribution contains information on both, the orientation and the colour of the edge, the correct structure is recovered (*right*).

### The coloured directed random graph with arbitrary degree distribution

To study the global behaviour of the polymer network, we are interested in quantities, such as the gel point (phase transition in the random graph), the gel fraction (relative size of the giant component), and the average molecular weight (expected size of all connected components). Here, we present the mathematical expressions for these quantities for the coloured directed random graph with arbitrary degree distribution.

In the coloured directed random graph^34^, every half-edge is assigned one colour $i=1,\ldots ,N$, according to the 2*N*-variate degree distribution. In- and out-edges are only paired if they have the same colour. Every node is characterised by a vector of half-edge counts $\mathbf{k}=(k_1,\dots ,k_{2N})$ of all possible half-edge types. The count $k_{2i-1}$ is the in-degree of colour *i* and $k_{2i}$ the out-degree of colour *i*. Thus, the degree distribution $u({\varvec{k}})$ defines the probability that a node is characterised by count vector ${\varvec{k}}$.

The pairing rules between the directed coloured half-edges are mathematically defined in permutation matrix ${\varvec{P}}$. If only one colour is present in the directed network ($N=1$), it is given by

1 $$\begin{aligned} {\varvec{P}}= \begin{pmatrix} 0 &{} 1 \\ 1 &{} 0 \end{pmatrix}=\varvec{\sigma }. \end{aligned}$$

A non-zero element $P_{i,j}=1$ indicates that a half-edge of type *i* pairs with a half-edge of type *j*. In the case of the uncoloured directed network, out-edges are paired with in-edges and the other way around, but two half-edges of the same type cannot be paired. In the case of *N* colours, the $2N\times 2N$ permutation matrix is given by

2 $$\begin{aligned} {\varvec{P}}= \left( \begin{array}{c c c c} \varvec{\sigma } &{} {\varvec{0}}&{}\dots &{}{\varvec{0}}\\ {\varvec{0}} &{} \varvec{\sigma } &{}\ddots &{}\vdots \\ \vdots &{}\ddots &{}\ddots &{}{\varvec{0}}\\ {\varvec{0}}&{}\dots &{}{\varvec{0}}&{}\varvec{\sigma } \end{array} \right) , \end{aligned}$$

with $\varvec{\sigma }$ as defined in Eq. (1) and ${\varvec{0}}$ being a $2\times 2$ zero matrix.

Some network properties in this model require to know only the first and second mixed moments of the degree distribution $u({\varvec{k}})$, and, in what follows, we simply write ${\mathbb {E}}[X]$ to denote expectation of some random variable *X* with respect to this distribution. We define the vector $\varvec{\mu }$ and matrix ${\varvec{M}}$ as

3 $$\begin{aligned} \mu _{i}={\mathbb {E}}[k_i],\;i=1,\dots ,2N, \end{aligned}$$

and

4 $$\begin{aligned} M_{i,j}=\frac{{\mathbb {E}}[k_i k_j]}{{\mathbb {E}}[k_j]}-\delta _{i,j}, \;i,j=1,\dots ,2N. \end{aligned}$$

The expectation values ${\mathbb {E}}[k_i]$ define the first partial moments of $u({\varvec{k}})$ and ${\mathbb {E}}[k_ik_j]$ the second partial moments. We consider degree distributions with finite partial moments.

In simple percolation, where each edge is removed with probability $1-p$, the percolation threshold^34^ $p_\text {crit}=\frac{1}{\lambda }$ is given by the solution of the following eigenvalue problem:

5 $$\begin{aligned} \varvec{PMv}=\lambda {\varvec{v}},\; \lambda \ge 1, \end{aligned}$$

where matrices ${\varvec{P}}$ and ${\varvec{M}}$ are as defined above. Here $p=p_\text {crit}$ indicates the point at which the giant component disappears under random removal of edges. However, the paradigm is different in the case of history-dependent networks where the degree distribution is time-dependant $u(\varvec{k},t)$. We wish to know at which point $t=t_\text {crit}>0$, matrix $\mathbf{M}(t)$, which continuously depends on time, starts to indicate existence of the giant component. Since for vanishing matrix we have $\lim \limits _{t\rightarrow 0}\det (\varvec{PM}(t)-{\varvec{I}})=1>0,$ such a point is given by the first sign change of the determinant:

6 $$\begin{aligned} t_\text {crit} = \inf \{t : \det (\varvec{PM}(t)-{\varvec{I}}) < 0\}, \end{aligned}$$

with ${\varvec{I}}$ being the identity matrix of size $2N\times 2N$. For $N=1$, Eq. (6) is equivalent to the criterion derived for uncoloured directed random graphs^21^.

In a similar manner as in Ref.^34^, we derive expressions for the fraction of nodes in the giant component and the weight average component size for the directed case, see “Methods” section for the detailed derivation. The fraction of nodes in the giant component $g_\text {node}$, that is the probability that a randomly sampled node is part of the giant component, is given by

7 $$\begin{aligned} g_\text {node}=1-{\mathbb {E}}[(\varvec{Ps})^{{\varvec{k}}}]. \end{aligned}$$

According to this definition, the actual number of nodes in the giant components is $g_\text {node}N_{sys}$, where $N_{sys}$ is the total number of nodes. The elements of vector ${\varvec{s}}=(s_1,\dots ,s_{2N})^\top$ are defined by

8 $$\begin{aligned} s_i=\frac{{\mathbb {E}}[k_i(\varvec{Ps})^{{\varvec{k}}-\varvec{e_i}}]}{{\mathbb {E}}[k_i]},\, i=1,\dots ,2N, \end{aligned}$$

with $\varvec{e_i}$ denoting the standard basis vectors and the vector power being evaluated as ${\varvec{s}}^{{\varvec{k}}}:=\prod _{i=0}^{N} s_i^{k_i}$. An expression for the weight-average size of finite components $\langle s \rangle _w$ is given by

9 $$\begin{aligned} \langle s \rangle _w=\frac{\langle n^2\rangle }{\langle n \rangle }=1+\frac{{\varvec{s}}^\top \varvec{DP}[{\varvec{I}}-{\varvec{X}}({\varvec{s}}){\varvec{P}}]^{-1}{\varvec{s}}}{1-g_\text {node}}, \end{aligned}$$

where ${\varvec{D}}=\text {diag}\{ {\mathbb {E}}[k_1],\dots ,{\mathbb {E}}[k_{2N}]\}$. The elements of the matrix function ${\varvec{X}}({\varvec{s}})$ are defined as

10 $$\begin{aligned} X_{i,j}({\varvec{s}})=\frac{{\mathbb {E}}[(k_i k_j-\delta _{i,j}k_i){\varvec{s}}^{{\varvec{k}}-\varvec{e_i}-\varvec{e_j}}]}{{\mathbb {E}}[k_i]}, \,i,j=1,\dots ,2N. \end{aligned}$$

## Random graph representation of chain-growth processes

**Figure 2:**
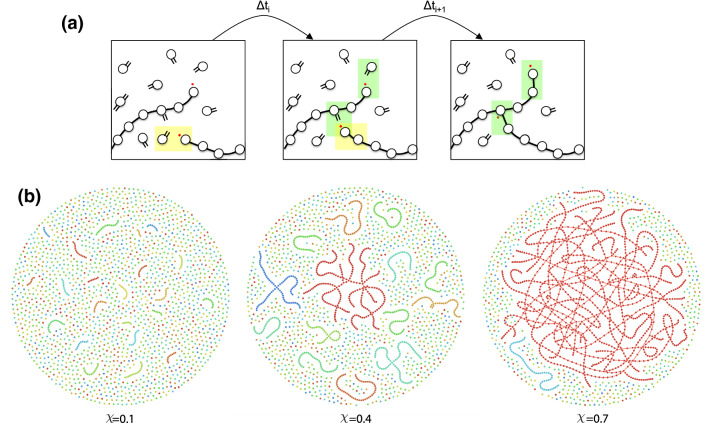
Cross-linked polymer network during chain-growth polymerisation. Illustration of (**a**) the dynamical process and (**b**) the snapshots of the evolving network. In (**a**) the red ‘$\cdot$’-symbol marks the active site and the ‘$=$’-symbol the functional groups. Three consecutive time instances are depicted with one bond formed in the first time interval (highlighted in yellow), and two bonds formed in the second (highlighted in green). In (**b**) the network topologies with system size $N_\text {sys}=2000$ are depicted for: $\chi =0.1$: early stage; $\chi =0.4$: close to the gel point; $\chi =0.7$: gel regime.

During chain-growth polymerisation, monomers join together and form long polymer chains or a polymer network as a result of chemical reactions. Such a linking process, as illustrated in Fig. 2a, takes place only at active sites that propagate from one monomer to the next by reacting with functional groups leaving a trace of covalently connected monomers behind. Since the propagation reaction is asymmetric, by which we mean that active (radical) sites react with functional groups, one may attribute a notion of direction to the resulting chemical bond. Let a directed edge be pointing from the former radical site to the former functional group, so that a monomer with the former radical obtains an out-edge, and the monomer with the former functional group an in-edge.

Depending on the number of functional groups on each monomer, the system forms linear polymer strands, sparsely cross-linked polymers, or densely cross-linked polymer networks. Chain-growth polymerisation follows a mechanisms, due to which *one* functional group is typically converted into *two* bonds. Therefore, a monomer with one functional group contributes to linear strands ($\deg =2$), whereas monomers with at least *two* functional groups act as crosslinks ($\deg =3,4$). Cross-linked systems may experience a phase transition from the soluble regime to the gel regime. After the transition, the gel further continues to undergo crosslinking and expands in its size. Figure 2b illustrates the structure of a cross-linked polymer system at different stages of its formation, before, close to, and after the gel point.

### Example: living polymerisation

In living polymerisation, the propagation is the main reaction responsible for growth of polymers, see Fig. 2a. As shown in the figure, during the propagation step an active site ($\cdot$) of one monomer reacts with the functional group ($=$) of a second monomer, the active site transfers (propagates) to the second monomer, and a covalent bond is formed. Here, we assume that $1\%$ of functional groups are active at any time, and that active sites are not being consumed.

We can predict global network properties from purely local information, such as: (1) the distribution of the number of neighbours of a monomer unit, (2) the formation time of the bonds, and (3) the directionality of the bond. This information comprises the coloured directed degree distribution, which is obtained from a system of ordinary differential equations (ODEs) implementing the reaction rate equations for monomers. Such models are given in “Mathematical description: living polymerisation of polymer chains” section for linear and “Mathematical description: free-radical photopolymerisation” section for cross-linked systems.

**Figure 3:**
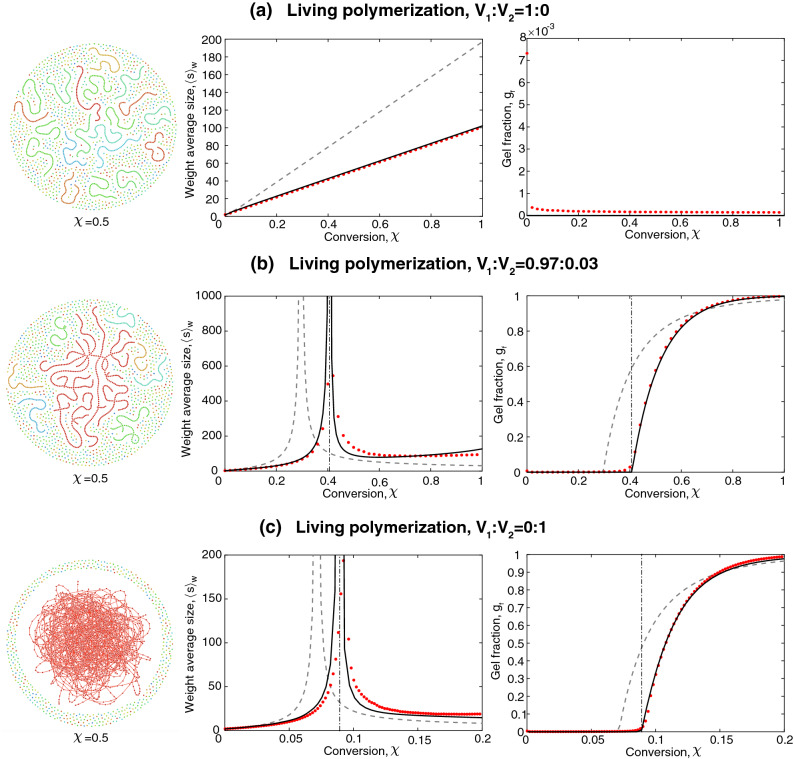
Network structure, average molecular weight, and gel fraction in living polymerisation: (**a**) linear polymers with $V_1:V_2=1:0$, (**b**) sparsely cross-linked polymers with $V_1:V_2=0.97:0.03$, (**c**) densely cross-linked polymers with $V_1:V_2=0:1$. The solid black line represents the coloured random graph with $N=35$, and the red data points represent the averaged results of 10 independent runs of SSA with $N_\text {sys}=10^6$. The dash-dotted line indicates the gel transition predicted by the coloured random graph. The network structures are obtained from SSA at conversion $\chi =0.5$.

We consider three different monomer mixtures: (a) monofunctional monomers $V_1:V_2=1:0$, (b) mixture of mono- and difunctional monomers $V_1:V_2=0.97:0.03$, (c) difunctional monomers $V_1:V_2=0:1$. In Fig. 3, we report the average size of the polymer molecules and the gel fraction (as given by Eqs. (7) and (9)) as a function of conversion (*i.e.*, the fraction of reacted functional groups) for the different polymerisation systems and compare these results to SSA. As a reference, predictions from a projected uncoloured directed random graph, which does not include information on the history of network formation, are depicted too.

If $N=1$, all edge pairs are equally likely to be linked. In the case of linear polymers, Fig. 3a, this allows for very short polymer chains, as well as very long ones, resulting in large variations in chain lengths. If the formation time is taken into account, the chain length distribution is additionally constrained and results in chains of similar length, as it should be in the case with linear living polymerisation.

Figure 3b,c shows that both systems, with $V_1:V_2=0.97:0.03$ and $V_1:V_2=0:1$, feature the phase transition. In both systems, the weight average size exhibits a singularity at the gel point. The gel fractions for the sparsely and densely cross-linked systems are shown in Fig. 3b (*right*) and 3b (*right*). Even though the gel point is shifted in both systems, the shift is significantly smaller in a more densely cross-linked system ($\Delta \chi \approx 0.02$) than in the sparsely cross-linked system ($\Delta \chi \approx 0.16$). This indicates that the history-dependence is less pronounced in networks with larger fractions of crosslinking points.

Overall, we observe good agreement between the coloured random graph with $N=35$ and the SSA with $N_\text {sys}=10^6$. Some minor deviations could be introduced by the small system size in the SSA.

### Example: free-radical photopolymerisation

As a second example, we consider free-radical polymerisation with photoinitiation. In contrast to living polymerisation, free-radical polymerisation allows the formation of new active sites (initiation) and also the consumption of active sites (termination) throughout the whole polymerisation process. Hence, chain length distributions in linear systems are typically broader than for living polymerisation. We aim to understand if the evolving structures still show history-dependence, and if so, to what extent.

The reaction scheme of free-radical polymerisation includes three competing reactions in the reaction kinetic ODEs model: (1) initiation, the transformation of a functional group into a radical, (2) propagation, the reaction between a radical with a functional group, resulting in a bond and transfer of the radical to the second monomer, and (3) termination, the consumption of two radicals by disproportionation (no bond formation). The initiation is induced by ultraviolet light irradiation, thus the term *photopolymerisation*.

The full kinetic model yielding the 2*N*-variate degree distribution for the entire polymerisation process is given in “Mathematical description: free-radical photopolymerisation” section. The kinetic rate parameters, and initial species concentrations in our study deviate from parameters in literature to enable a comparison with SSA, see Table 1. In real experimental conditions, the radical concentration is typically lower: the monomer fraction carrying a radical is of the order of $10^{-5} {-}10^{-7}$. Such values are easy to account for in our model, but are hard to achieve in SSA that requires large species concentrations. Thus for the sake of comparison, we: (1) adjusted the reaction rates and increased the rate coefficients for photoinitiation and termination $\frac{k_\text {d}}{k_\text {t}}$, and (2) choose the initiator concentration to be such that it is not depleted during the polymerisation process.

**Table 1:** Kinetic parameters and initial concentrations (not representative for real-world systems).

Symbols	Values	SI units	Definitions
$k_\text {d}$	1.30	$\text {s}^{-1}$	Rate constant photoinitiation
$k_\text {ini}$	$6.12\times 10^4$	$\text {l} (\text {mol}\, \text {s})^{-1}$	Rate constant vinyl initiation
$k_\text {p}$	$6.12\times 10^4$	$\text {l} (\text {mol}\, \text {s})^{-1}$	Rate constant propagation
$k_\text {td}$	$4.40\times 10^5$	$\text {l} (\text {mol}\, \text {s})^{-1}$	Rate constant termination
$I_2(0)$	1.03	$\text {mol}\, \text {l}^{-1}$	Initial concentration of photoinitiator
*I*(0)	0	$\text {mol}\, \text {l}^{-1}$	Initial concentration of photoinitiator
${\mathcal {M}}_0$	1	$\text {mol}\, \text {l}^{-1}$	Initial concentration of monomers
$R_0$	$0.01,\,0.1$	–	Initial fraction of radicals
$c_\text {mono}$	$1,\,0.01,\,0$	–	Initial fraction of monofunctional monomers
$c_\text {di}$	$0,\,0.01,\,1$	–	Initial fraction of difunctional monomers

**Figure 4:**
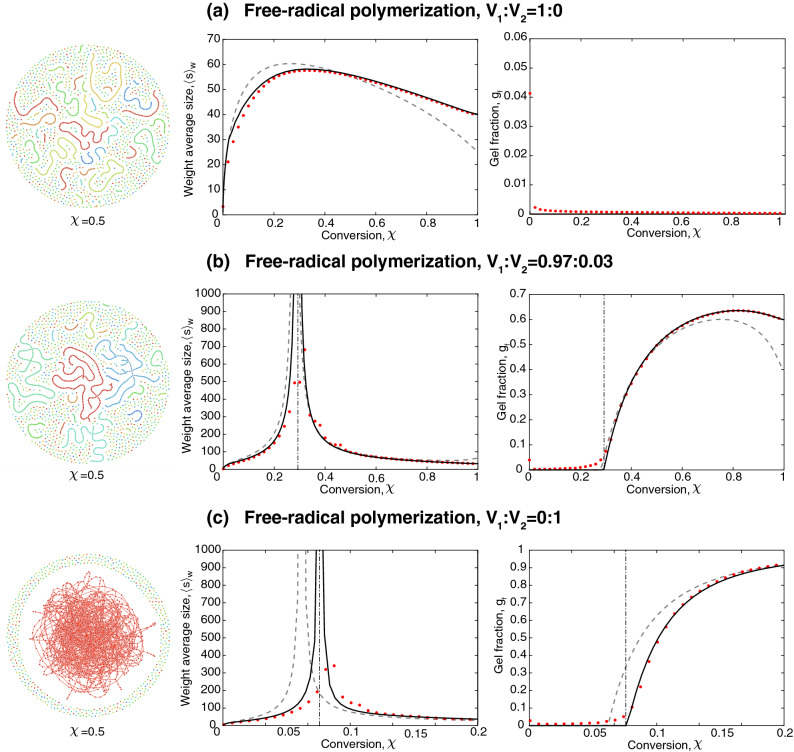
Network structure, average molecular weight, and gel fraction in free-radical polymerisation: (**a**) linear polymers with $V_1{:}V_2=1{:}0$, (**b**) sparsely cross-linked polymers with $V_1:V_2=0.97:0.03$, (**c**) densely cross-linked polymers with $V_1{:}V_2=0{:}1$. The solid black line presents the results from the coloured random graph with $N=40$, and the red data points represents the averaged results of 10 independent SSA runs with system size $N_\text {sys}=10^6$. The dash-dotted line indicates the gel transition predicted by the coloured directed random graph. The network structures are obtained with SSA at conversion $\chi =0.5$.

We study the polymerisation of mixtures of mono- and divinyl monomers. In Fig. 4, the weight average size, the gel fraction and network structures are depicted for three polymerisation systems with varying ratio of mono- and divinyl monomers $V_1{:}V_2$: (a) linear chains $V_1{:}V_2=1{:}0$, (b) sparsely cross-linked network $V_1{:}V_2=0.97{:}0.03$, (c) densely cross-linked network $V_1{:}V_2=0{:}1$. The predictions of the coloured directed random graph is compared to the SSA data and the uncoloured directed random graph.

The structure of the linear system $V_1{:}V_2=1{:}0$ confirms that chains of various sizes are present in the system simultaneously, which differs from the structure obtained from living polymerisation (Fig. 3). Also the network structure of the sparsely cross-linked system with $V_1:V_2=0.97{:}0.03$ shows a broader range of molecular sizes. The densely cross-linked network $V_1{:}V_2=0{:}1$ looks similar to the network obtained from living polymerisation as in both cases the gel fraction is close to 1. Nevertheless, in free-radical polymerisation new polymer molecules are formed by the initiation of free monomers throughout the polymerisation process, but they quickly get incorporated into the gel.

Figure 4a, (*left*) depicts the weight average size for the linear system. The predictions of the uncoloured versus the coloured random graph diverge less strongly than in the case of living polymerisation, see Fig. 3a (*left*). This is due to the continuous formation and termination of radicals throughout the polymerisation process and their hence limited life-time, which reflects in a large variability of the chain length. The calculated length distributions are broader than in the case of living polymerisation and are more similar to distributions obtained from the uncoloured directed random graph. Hence, the uncoloured model gives better predictions of the weight average size for free-radical polymerisation than for living polymerisation.

Figure 4b,c, illustrate the weight average size and the gel fraction in a sparsely and densely cross-linked polymer networks. In a sparsely cross-linked system, labelling edges with their formation time does not significantly alter the predictions of the gel point and the weight average size. However, it does improve the predictions of the gel fraction at large conversions, $\chi >0.6$. In our example of a densely cross-linked system, the uncoloured random graph underestimates the gel point by $\Delta \chi \approx 0.02$. The history-dependence is less pronounced in free-radical polymerisation when compared to living polymerisation, but also within living polymerisation the extent of this effect varies across the parameter space.

### Quantification of the history-dependency

**Figure 5:**
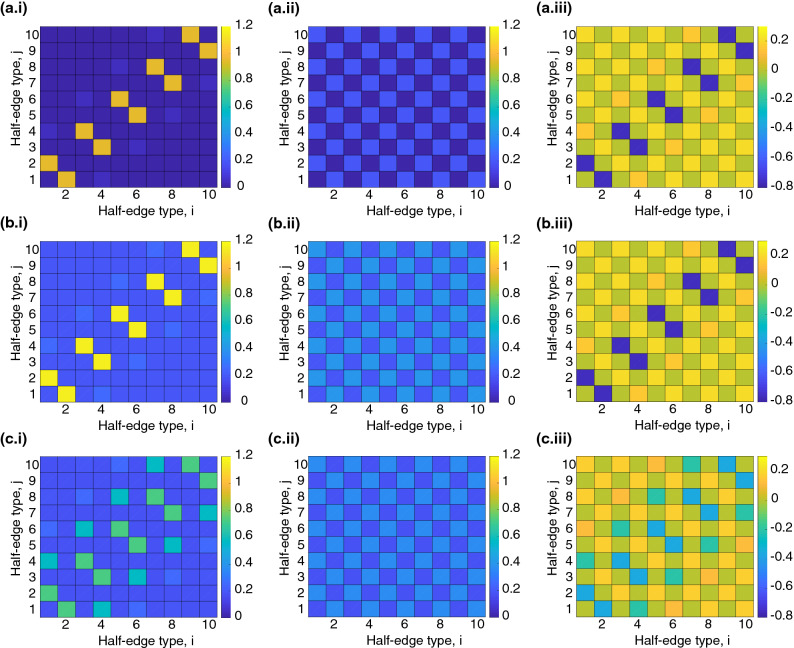
Examples of ${\varvec{M}}$-matrices for different systems with $N=5$ colours: (**a**) living polymerisation with $V_1:V_2=1:0$ and $p_{ini}=0.01$, (**b**) living polymerisation with $V_1:V_2=0:1$ and $p_{ini}=0.01$, (**c**) living polymerisation with $V_1:V_2=0:1$ and $p_{ini}=0.1$. The following matrices are depicted: (i) ${\varvec{M}}$ according to Eq. (4), (ii) $\tilde{{\varvec{M}}}$ according to Eq. (43), (iii) ${\varvec{M}}_\text {diff}=\tilde{{\varvec{M}}}-{\varvec{M}}$.

We use ${\varvec{M}}$ obtained at full conversion, as it contains information of the entire polymerisation process. If $M_{i,j}$ is close to zero, the half-edges of types *i* and *j* are rarely found on one monomer simultaneously. Let $\tilde{{\varvec{M}}}$ corresponds to a system with randomised colours, a system with ’erased’ history-dependence. Figure 5 shows structures of ${\varvec{M}}$ and $\tilde{{\varvec{M}}}$ for: (a) linear living polymerisation with initial radical concentration $p_\text {ini}=0.01$, (b) densely cross-linked living polymerisation with initial radical concentration $p_\text {ini}=0.01$, (c) densely cross-linked living polymerisation with increased initial radical concentration $p_\text {ini}=0.1$.

The first column (i) illustrates the matrix elements of ${\varvec{M}}$ for the fully polymerised systems $\chi =1$. In linear systems, only two distinct half-edge types can be present simultaneously on one node. We only observe significant values in the diagonal band $M_{2(i-1),2i}$ and $M_{2i,2(i-1)}$ with $i=1,\dots ,N$. Hence, the two half-edges present on the same node are likely to be of same colour and opposite orientation. This is a consequence of the chosen polymerisation process: Only rarely do time intervals change within the characteristic time of a propagation step. All other matrix elements are small since: (1) radicals do not remain on the same node for more than one time interval, and (2) it is not possible that two in- or two out-edges are present on the same node as every monomer has only one vinyl group.

Figure 5b.i presents ${\varvec{M}}$ for densely cross-linked systems and the initial radical concentration of $p_\text {ini}=0.01$. Initially, every monomer has two vinyl groups leading to a maximum degree $\deg =4$. The consumption of the two vinyl groups during polymerisation is independent. The overall pattern is similar to (a.i) with a non-zero offset; all combinations of half-edge types are allowed in the system: Even though the colours of the in- and out-edges formed by one vinyl site are correlated, one active site may be consumed in the beginning of the process whereas the other site is consumed in the end. Typically, at full conversion a node has more than one half-edge of given orientation (two in-edges and two out-edges) and the half-edges of the same orientation are likely to have different colours.

Figure 5c.i shows ${\varvec{M}}$ for densely cross-linked living polymerisation with an increased initial radical concentration, $p_\text {ini}=0.1$. The observed pattern is different from the previous cases (a,b.i) as it shows a broader band of large matrix elements. The observed trend is caused by two effects: (1) The more radicals are present in the system, the more nodes with one in-edge of colour *i* and one out-edge of colour $i+1$ are formed when the time interval changes. Thus, the larger the radical concentration, the larger are the elements $M_{2(i-1),2(i+1)}$ and $M_{2(i+1),2(i-1)}$ with $i=1,\dots ,N-1$. (2) As polymerisation happens faster in systems with larger radical concentration and the number of time intervals is fixed and equally spaced over conversion, the absolute values $\Delta t_i$ are shorter. Hence, radicals are more probable to remain on one monomer for more than one time interval.

To quantify the extent of the history-dependence of a system, we compare its ${\varvec{M}}$-matrix to the matrix $\tilde{{\varvec{M}}}$ with randomised colours, which represents a system with ’erased’ history-dependence. The randomised system satisfies the same degree distribution with respect to the orientation of the edge, but with a random distribution of edge colours. The derivation of $\tilde{{\varvec{M}}}$ is given in “[Sec Sec18]” section. If ${\varvec{M}}=\tilde{{\varvec{M}}}$, the observed network is not history-dependent and can be sufficiently described by the uncoloured random graph, i.e., colours are redundant. One example of such a process is the conventional step-growth polymerisation^24^.

We now quantify the degree of history-dependence using the following score:

11 $$\begin{aligned} h=\frac{||{\varvec{M}}-\tilde{{\varvec{M}}}||_F}{|| \tilde{{\varvec{M}}} ||_F}. \end{aligned}$$

**Figure 6:**
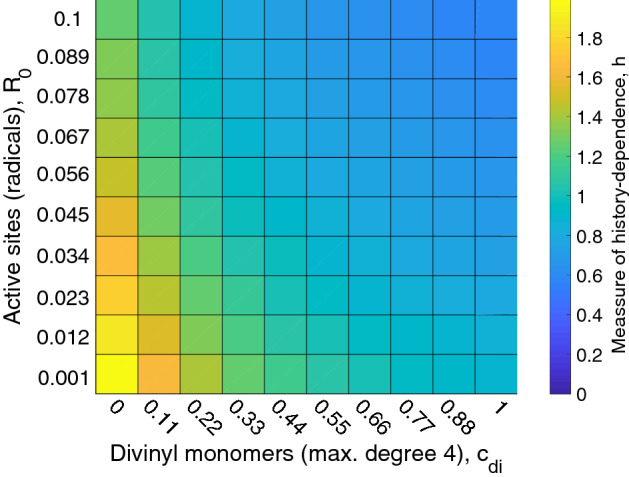
History-dependence in living polymerisation. The measure of history-dependence for different divinyl and radical fractions. The depicted *h*-value is obtained for systems with $N=5$ colours.

**Figure 7:**
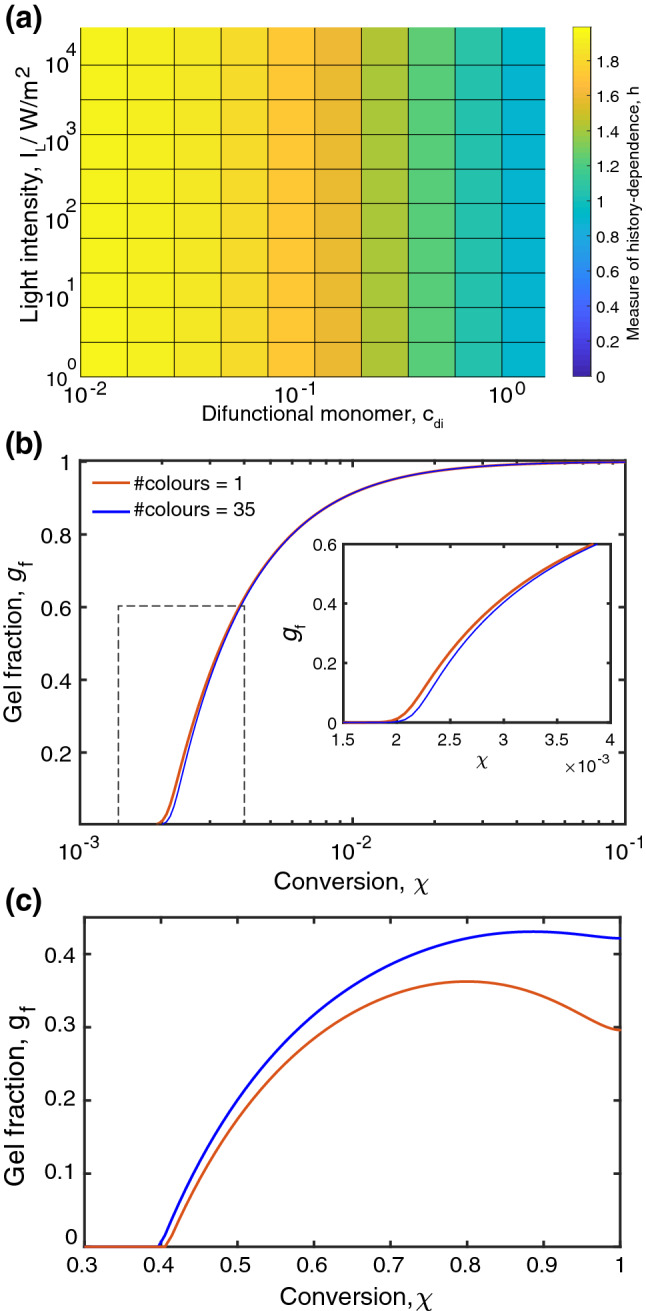
Photo-initiated polymerisation. The coloured model for free-radical polymerisation supplemented with parameters used in acrylate coatings^13^. This process is initiated and controlled by light irradiation with constant intensity $I_\text {L}$, which produce radicals, see Methods for details. (**a**) History-dependence is sensitive to the fraction $c_\text {di}$ of difunctional monomer but not to light intensity. (**b**) Gel fraction buildup at $I_\text {L}=1,000\, \text {W}/\text {m}^2$ and $c_\text {di}=1$ features weak history dependency, wheres for $c_\text {di}=0.1$ history dependency becomes more pronounced, as shown in (**c**).

The entry-wise matrix norm is defined as $|| {\varvec{M}} ||_F:=\left( \sum _{i,j} |M_{i,j}|^2\right) ^{1/2}$. The randomised matrices for the systems presented in Fig. 5 are illustrated in column (ii). For the system of linear living polymerisation (Fig. 5a.ii) we obtain a checkerboard pattern with zero elements for correlations of two half-edges of equal orientation $M_{2(i-1),2(j-1)}$ and $M_{2i,2j}$ for $i,j=1,\dots ,N$. Non-zero elements are obtained for two half-edge types with opposite orientation, all of equal value. For densely cross-linked living polymerisation we observe a similar checkerboard pattern, however with all elements shifted to larger values and without zero elements. In contrast to ${\varvec{M}}$, the randomised matrix $\tilde{{\varvec{M}}}$ is to a large extent independent of the initial radical concentration, see Fig. 5b.ii,c.ii. These trends are summarised in Fig. 6, where we plot the measure of the history-dependence *h* as a function of two parameters: the fraction of active sites (radicals) and the fraction of crosslinkers (divinyl monomers). An increased fraction of radicals results in a decrease of the *h*-value, implying that networks formed with high radical concentrations exhibit less history-dependence: as the polymerisation process becomes ‘less sequential’ and hence closer resembling to the step-growth polymerisation. Conversely, the effect of the history-dependence in sparsely cross-linked systems is more pronounced than in densely cross-linked ones. Figure 7, show that in photo-initiated polymerisation, where light irradiation is used to control the radical concentration, the divinyl monomer fraction is the main parameter determining degree of history dependance.

## Conclusion

Networks produced by chain-growth polymerisation are constrained to form in a sequential manner and thus differ from networks resulting from the step-growth process. Such a difference results in the history-dependence of the network structure: If the network were to be cut into separate monomers and then rewired randomly by maintaining their degree sequence, a very different network would be obtained. This work shows that the structure of such history-dependent networks corresponds to *coloured* random graphs that solely rely on local description of the monomers. This association between polymers and random graphs enables analytical methods and fast algorithms for studying the structure of polymer networks originated from the chain-growth mechanism. We expect that such a fundamental connection to random graphs could be exploited to facilitate design and optimisation of polymer materials. The objectives for design depend on particular application. For example, practitioners designing polymer recipes may be interested to either avoid gelation while maximising molecular weight when controlling viscosity of a polymer melt, or to maximise gel fraction to achieve stronger materials, as in coating and thin film production. Another example is volumetric three-dimensional printing^35–39^, where the objective is to maximise steepness of the gel fraction curve, which guaranties high contrast of printed objects.

By computing a score function, we show that only low radical concentration and small fraction of difunctional monomers was found to be associated with significant history-dependency. Coincidently the region of the parameter space is often exploited in polymer design to enhance elasticity^37,40–42^, whereas the region is also poorly tractable with conventional SSA, which has the best performance when concentrations of all species are comparable^43^.

Not all polymerisation process lead to pronounced history-dependency, and hence they may be satisfactory represented with simple random graphs without colours. Even in extreme cases of industrially relevant processes with history-dependency that we have studied (living and free radical polymerisation) the discrepancy between gel fraction curves was found to be small comparing to what can be achieved with altering monomer functionality. We thus hypothesise that polymer networks that are currently produced exploit a rather small subset of structural forms that can be possibly accommodated by coloured random graphs.

## Methods

### Derivation of the size of the giant component

Let us consider a system of nodes bearing 2*N* different types of half-edges. The probability that a node is characterised by the half-edge count vector ${\varvec{k}}=(k_1,\dots ,k_{2N})$ is given by the multivariate degree distribution $u({\varvec{k}})$. The permutation matrix *P* defines a pairing on the 2*N* half-edge types, with $P_{i,j}=1$ if half-edge types *i* and *j* form a bond and $P_{i,j}=0$ otherwise.

Let the *i*-excess degree distribution $u_i({\varvec{k}})$ be the probability that a node connected to a randomly sampled half-edge of type *i* has excess configuration ${\varvec{k}}$ (thus not counting the already sampled half-edge). It is given by

12 $$\begin{aligned} u_i({\varvec{k}})=(k_i+1)\frac{u({\varvec{k}}+\varvec{e_i})}{{\mathbb {E}}[k_i]}, \end{aligned}$$

where $\varvec{e_i}$ are the standard basis vectors.

Let ${\varvec{z}}=(z_1,\dots ,z_{2N}),\;|z_i|<1,\; z_i\in \mathbb C$, we define the generating functions of the degree distribution

$$\begin{aligned} U({\varvec{z}}): {\mathbb {C}}^{2N} \rightarrow {\mathbb {C}} \end{aligned}$$

and the *i*-excess degree distribution

$$\begin{aligned} U_i({\varvec{z}}): {\mathbb {C}}^{2N} \rightarrow {\mathbb {C}} \end{aligned}$$

as

13 $$\begin{aligned} U({\varvec{z}})&=\sum _{{\varvec{k}}>0} {\varvec{z}}^{{\varvec{k}}} u({\varvec{k}}),\\ U_i({\varvec{z}})&=\sum _{{\varvec{k}}>0} {\varvec{z}}^{{\varvec{k}}} u_i({\varvec{k}})=\frac{1}{{\mathbb {E}}[k_i]}\frac{\partial U({\varvec{z}})}{\partial z_i}, \;i=1,\dots ,2N \end{aligned}$$

Note that the vector power in these definitions and in what follows is evaluated as

$$\begin{aligned} {\varvec{z}}^{{\varvec{k}}}:=\prod _{i=0}^{2N} z_i^{k_i}. \end{aligned}$$

Let $W(x): {\mathbb {C}} \rightarrow {\mathbb {C}}$ and $W_i(x): {\mathbb {C}} \rightarrow {\mathbb {C}}$ for $i=1,\dots 2N$ be a family of complex functions satisfying the system of $2N+1$ equations:

14 $$\begin{aligned}{}& W(x)=xU({\varvec{P}}\varvec{\omega }(x)),\\&W_i(x)=xU_i({\varvec{P}}\varvec{\omega }(x)),\,i=1,\dots ,N \end{aligned}$$

with $\varvec{\omega }(x):=\left( W_1(x),\dots ,W_{2N}(x)\right) ^\top$. Here $W_i(x)$ play an auxiliary role as we are mainly concerned with the properties of *W*(*x*). The theory for coloured random graphs^34^ interprets *W*(*x*) as the generating function for the probability *w*(*n*) that a uniformly at random chosen node is part of a finite component of size *n*, that is $W(x)=\sum _{n>0} x^{n} w(n)$ with $x\in {\mathbb {C}},\,|x|\le 1.$ Therefore, the probability that a randomly sampled node is not in any finite-sized component but in the giant component is given by

15 $$\begin{aligned} g_\text {node}=1-W(1), \end{aligned}$$

where *W*(1) can be calculated by substituting $x=1$ in Eq. (14), yielding

16 $$\begin{aligned} W(1)&=\sum _{{\varvec{k}}>0} u({\varvec{k}}) (\varvec{Ps})^{{\varvec{k}}}={\mathbb {E}}[(\varvec{Ps})^{{\varvec{k}}}],\; \end{aligned}$$

with ${\varvec{s}}=\varvec{\omega }(1)$, satisfying system of equations

17 $$\begin{aligned} s_i&=W_i(1)=U_i(\varvec{Ps})\\&=\sum _{{\varvec{k}}>0} u_i({\varvec{k}}) (\varvec{Ps})^{{\varvec{k}}}\\&=\frac{1}{{\mathbb {E}}[k_i]} \sum _{{\varvec{k}}>0} (k_i+1) u(k_i+\varvec{e_i}) (\varvec{Ps})^{{\varvec{k}}}\\&=\frac{{\mathbb {E}}[k_i(\varvec{Ps})^{{\varvec{k}}-\varvec{e_i}}]}{{\mathbb {E}}[k_i]}, \end{aligned}$$

for $i=1,\dots ,2N$. These results were stated in Eqs. (7) and (8).

### Derivation of the weight-average size of finite components

We derive the expression of the weight-average size of finite components, defined as

18 $$\begin{aligned} \langle s \rangle _w=\frac{\frac{d}{dx}W(x)|_{x=1}}{1-g_\text {node}}, \end{aligned}$$

and normalised to the system size without the giant component. The evaluation of $\frac{d}{dx}W(x)$ results in

19 $$\begin{aligned} \frac{dW(x)}{dx} \bigg |_{x=1}&=U(\varvec{P\omega }(x))|_{x=1}+\\&\quad+\bigg (\frac{\partial U({\varvec{z}})}{\partial z_1} \frac{d(\varvec{P\omega }(x))_1}{dx} + \dots \\&\quad+\frac{\partial U({\varvec{z}})}{\partial z_N} \frac{d(\varvec{P\omega }(x))_N}{dx} \bigg )\bigg |_{x=1,{\varvec{z}}={\varvec{s}}}\\&=W(1)+\left( \frac{\partial U({\varvec{z}})}{\partial z_1},\dots ,\frac{\partial U({\varvec{z}})}{\partial z_N}\right) \bigg |_{{\varvec{z}}={\varvec{s}}}{\varvec{P}}{\varvec{y}}\\&=1-g_\text {node}+{\varvec{s}}^\top {\varvec{D}}{\varvec{P}}{\varvec{y}}. \end{aligned}$$

with ${\varvec{y}}=\frac{d}{dx}{\varvec{w}}(x)|_{x=1}$ and ${\varvec{D}}=\text {diag}\{{\mathbb {E}}[k_1],\dots ,{\mathbb {E}}[k_{2N}]\}$. The elements of vector ${\varvec{y}}=(y_1,\dots ,y_N)^\top$, where $y_i=\frac{\partial }{\partial x}W_i(x)|_{x=1}$, are given by

20 $$\begin{aligned} y_i&=U_i(\varvec{P\omega }(x))|_{x=1}+\\&\quad+\bigg (\frac{\partial U_i({\varvec{z}})}{\partial z_1} \frac{d(\varvec{P\omega }(x))_1}{dx} + \dots \\&\quad+\frac{\partial U_i({\varvec{z}})}{\partial z_N} \frac{d(\varvec{P\omega }(x))_N}{dx} \bigg )\bigg |_{x=1,{\varvec{z}}={\varvec{s}}}\\&=s_i+\left( \frac{\partial U_i({\varvec{z}})}{\partial z_1},\dots ,\frac{\partial U_i({\varvec{z}})}{\partial z_N}\right) \bigg |_{{\varvec{z}}={\varvec{s}}}{\varvec{P}}{\varvec{y}}. \end{aligned}$$

Utilising the matrix function ${\varvec{X}}({\varvec{s}})$ with its elements

21 $$\begin{aligned} X_{i,j}({\varvec{s}})&=\frac{\partial U_i({\varvec{z}})}{\partial {z_j}}\bigg |_{{\varvec{z}}={\varvec{s}}}\\&=\frac{{\mathbb {E}}[(k_ik_j-\delta _{i,j}k_i){\varvec{s}}^{{\varvec{k}}-\varvec{e_i}-\varvec{e_j}}]}{{\mathbb {E}}[k_i]}, \end{aligned}$$

with $i,j=1,\dots ,2N$, Eq. (20) is written in its matrix form ${\varvec{y}}={\varvec{s}}+{\varvec{X}}({\varvec{s}})\varvec{Py}$. Thus, ${\varvec{y}}$ is expressed explicitly as

22 $$\begin{aligned} {\varvec{y}}&=[{\varvec{I}}-\varvec{XP}({\varvec{s}})]^{-1}{\varvec{s}}. \end{aligned}$$

Having Eqs. (19) and (22) in hand, Eq. (18) is written as

23 $$\begin{aligned} \langle s \rangle _w=1+ \frac{{\varvec{s}}^\top {\varvec{D}}{\varvec{P}}[{\varvec{I}}-\varvec{XP}({\varvec{s}})]^{-1}{\varvec{s}}}{1-g_\text {node}}, \end{aligned}$$

which is stated in Eq. (9).

### Mathematical description: living polymerisation of polymer chains

In this section, we introduce a master equation for monomer species in living polymerisation. First we will discuss the formulation without colours, followed by the monomer PBE with *N* colours. Living polymerisation is a special type of chain-growth polymerisation characterised by a very fast initiation and suppressed termination of radicals. Hence, only propagation is modelled explicitly. We consider a monomer with one functional group (vinyl group), which allows the formation of at maximum two bonds per monomer and therefore leads to linear strands of polymers.

#### Monomer equation for living chain polymerisation

The monomer equation describes the concentration of the *monomer* species over time by a system of ODEs, rather than the concentrations of polymers of different size as is the case with the conventional PBE. We consider a system with the initial fraction of active monomers units to be set to $R_0=0.01$. During the propagation reaction, the active monomer unit connects with an unreacted monomer and a covalent bond is formed. Due to the asymmetry in the reactants (vinyl+radical), the formed connection is considered asymmetric as well. This asymmetry is represented as the orientation of a directed edge. Monomer species $\mathcal M_{k_1,k_2}$ are characterised by the number and direction of edges they bear—the number of in-edges $k_1$ and out-edges $k_2$.

In order to describe living polymerisation, we need to distinguish between five monomer species: (1) free monomers ${\mathcal {M}}_{0,0}$, (2) active monomers ${\mathcal {M}}^*_{0,0}$ with zero (half-) edges, (3) active monomers ${\mathcal {M}}_{1,0}$ with one in-edge and zero out-edges, (4) dead ends ${\mathcal {M}}_{0,1}$, which initially were active sites and have already connected to one monomer with zero in-edges and one out-edge, and (5) consumed monomers $\mathcal M_{1,1}$ with one in-edge and one out-edge. The polymerisation process is described by the following reaction mechanism:

24 $$\begin{aligned}&{\mathcal {M}}^*_{0,0}+{\mathcal {M}}_{0,0}\xrightarrow {k_\text {p}}{\mathcal {M}}_{0,1}+{\mathcal {M}}_{1,0},\\&{\mathcal {M}}_{1,0}+{\mathcal {M}}_{0,0}\xrightarrow {k_\text {p}}\mathcal M_{1,1}+{\mathcal {M}}_{1,0}. \end{aligned}$$

Active monomer units ${\mathcal {M}}^*_{0,0}$ or ${\mathcal {M}}_{1,0}$ connect to free monomers ${\mathcal {M}}_{0,0}$ at rate $k_\text {p}$. The product species consist of one consumed monomer $\mathcal M_{0,1}$ or ${\mathcal {M}}_{1,1}$ and a new active monomer $\mathcal M_{1,0}$. This reaction mechanism translates into the following system of ODEs for monomer concentrations, which we denote as ${\mathcal {M}}_{i,j}(t)$:

25 $$\begin{aligned}&\dot{{\mathcal {M}}}_{0,0}(t)= -k_\text {p} {\mathcal {M}}_{0,0}(t) [{\mathcal {M}}_{1,0}(t) + {\mathcal {M}}^*_{0,0}(t) ], \\&\dot{{\mathcal {M}}}^*_{0,0}(t)= -k_\text {p} {\mathcal {M}}_{0,0}(t) {\mathcal {M}}^*_{0,0}(t), \\&\dot{{\mathcal {M}}}_{0,1}(t)= k_\text {p} {\mathcal {M}}_{0,0}(t) {\mathcal {M}}^*_{0,0}(t),\\&\dot{{\mathcal {M}}}_{1,0}(t)= k_\text {p} {\mathcal {M}}_{0,0}(t) {\mathcal {M}}^*_{0,0}(t),\\&\dot{{\mathcal {M}}}_{1,1}(t)=k_\text {p} {\mathcal {M}}_{0,0}(t) \mathcal M_{1,0}(t), \end{aligned}$$

with the initial conditions being set as follows: $\mathcal M_{0,0}(0)={\mathcal {M}}_0(1-R_0)$, ${\mathcal {M}}^*_{0,0}(0)=\mathcal M_0R_0$, ${\mathcal {M}}_{0,1}(0)=0$, ${\mathcal {M}}_{1,0}(0)=0$ and ${\mathcal {M}}_{1,1}(0)=0$.

We quantify the progress of the polymerisation by the conversion of functional groups, $\chi (t)$, which, in the case of linear polymers, is equivalent to the conversion of monomers:

26 $$\begin{aligned} \chi (t)=1-\frac{{\mathcal {M}}(t)}{{\mathcal {M}}(0)}. \end{aligned}$$

The two-variate degree distribution $u(k_1,k_2)$ at any time point *t* is extracted from the solution of Eq. (25) by setting:

27 $$\begin{aligned} u(k_1,k_2)=\frac{{\mathcal {M}}_{k_1,k_2}(t)+\mathcal M^*_{k_1,k_2}(t)}{\sum _{k_1,k_2}[{\mathcal {M}}_{k_1,k_2}(t) +\mathcal M^*_{k_1,k_2}(t)]}. \end{aligned}$$

#### ODEs with colours for living chain polymerisation

We extend the system of ODEs from the previous section to incorporate the formation time of edges in the species description, which allows to compute the coloured degree distribution. Consider *N* time intervals $\Delta t_c=t_{c+1}-t_c$ with $c=1,\dots ,N$. To each newly formed edge we assign colour *c* if its formation time $t\in [t_{c-1},t_c)$. The monomer species $\mathcal M_{{\varvec{k}}}$ are now indexed by the count vector

28 $$\begin{aligned} {\varvec{k}}=(k_1,\dots ,k_{2N}), \end{aligned}$$

where the index $k_{2(i-1)+1}$ denotes the count of in-edges of colour *i* and $k_{2i}$ the count of out-edges of colour *i*, for $i=1,\dots ,N$. In the case of linear polymer chains, the count vector elements are restricted to $k_{2(i-1)+1},\,k_{2i}\in \{0,1\}$, which does not hold true for a polymer network. We group the count vectors by the total number of in-edges and out-edges irrespectively of their colour:

$$\begin{aligned} {\varvec{k}}_{m,n}=\{ {\varvec{k}} : \sum _{i=1}^N k_{2(i-1)+1} =m, \; \sum _{i=1}^N k_{2i}=n \}. \end{aligned}$$

Let the elements of vectors $\Delta {\varvec{k}}_{1,0}(c)$ and $\Delta {\varvec{k}}_{0,1}(c)$ be given by

29 $$\begin{aligned}&[\Delta {\varvec{k}}_{1,0}(c)]_i=\delta _{i,2(c-1)+1},\\&[\Delta {\varvec{k}}_{0,1}(c)]_i=\delta _{i,2c},\\ \end{aligned}$$

with $\delta _{i,j}$ being Kronecker delta, and let

$$\begin{aligned} \Theta (t,c)= [\theta (t-t_c)+\theta (t_{c+1}-t)], \end{aligned}$$

with $\theta (t)$ the Heaviside step function. For all $c=1,\dots ,N$ representing time intervals, the reaction mechanism of Eq. (24) is extended using the multi-index notation:

30 $$\begin{aligned}&{\mathcal {M}}^*_\mathbf{0}+{\mathcal {M}}_{ \mathbf{0}}\xrightarrow {k_\text {p}} {\mathcal {M}}_{ \Delta {\varvec{k}}_{0,1}(c)} + {\mathcal {M}}_{\Delta {\varvec{k}}_{1,0}(c)},\\&{\mathcal {M}}_{{\varvec{k}}}+{\mathcal {M}}_{ \mathbf{0}}\xrightarrow {k_\text {p}} {\mathcal {M}}_{{\varvec{k}+\Delta {\varvec{k}}_{0,1}(c)}} + {\mathcal {M}}_{ \Delta {\varvec{k}}_{1,0}(c)},\;{{\varvec{k}}}\in {\varvec{k}}_{1,0} \end{aligned}$$

The latter reaction mechanism translates into a system of ODEs as follows. For monomer species that have no half-edges, we have:

31 $$\begin{aligned} \dot{{\mathcal {M}}}_\mathbf{0}(t)&= -k_\text {p} {\mathcal {M}}_\mathbf{0}(t)\left[ {\mathcal {M}}^*_{ \mathbf{0}}(t) + \sum _{ \varvec{\kappa }\in {\varvec{k}}_{1,0}} {\mathcal {M}}_{ \varvec{\kappa }}(t)\right] ,\\ \dot{{\mathcal {M}}}^*_{ \mathbf{0}}(t)&=-k_\text {p} {\mathcal {M}}_\mathbf{0}(t) {\mathcal {M}}^*_{ \mathbf{0}}(t),\\ \end{aligned}$$

For monomers with one out-edge of arbitrary colour, we have

32 $$\begin{aligned} \dot{{\mathcal {M}}}_{{\varvec{k}}}(t)=k_\text {p} {\mathcal {M}}_\mathbf{0}(t) {\mathcal {M}}^*_\mathbf{0}(t)\Theta (t,c), \end{aligned}$$

for all ${{\varvec{k}}} \in {\varvec{k}}_{0,1}$. For monomer species with one in-edge of arbitrary colour, we have:

33 $$\begin{aligned} \dot{{\mathcal {M}}}_{{\varvec{k}}}(t)=&-k_\text {p} {\mathcal {M}}_\mathbf{0}(t) {\mathcal {M}}_{{\varvec{k}}}(t) \\&+ k_\text {p}{\mathcal {M}}_\mathbf{0}(t) \left[ {\mathcal {M}}^*_\mathbf{0}(t) + \sum _{\varvec{\kappa }\in {\varvec{k}}_{1,0}} {\mathcal {M}}_{ \varvec{\kappa }}(t) \right] \Theta (t,c), \end{aligned}$$

for all ${\varvec{k}} \in {\varvec{k}}_{1,0}$. For monomer species with one in-edge of arbitrary colour and one out-edge of colour *c* we have:

34 $$\begin{aligned} \dot{{\mathcal {M}}}_{{\varvec{k}}+\Delta {\varvec{k}}_{0,1}(c)}(t)= k_\text {p} {\mathcal {M}}_\mathbf{0}(t) {\mathcal {M}}_{{\varvec{k}}}(t) \Theta (t,c), \end{aligned}$$

for all ${\varvec{k}}\in {\varvec{k}}_{1,0}$. The initial conditions are given by ${\mathcal {M}}_\mathbf{0}(0)={\mathcal {M}}_0(1-R_0)$, ${\mathcal {M}}^*_\mathbf{0}(0)={\mathcal {M}}_0R_0$ and ${\mathcal {M}}_{ {\varvec{k}}}(0)=0$ for ${\varvec{k}} \ne \mathbf{0}$.

From the solution of the ODE system (29)–(34), the 2*N*-variate degree distribution at time *t* is computed:

35 $$\begin{aligned} u({\varvec{k}})=\frac{{\mathcal {M}}_{{\varvec{k}}}(t)+\mathcal M^*_{{\varvec{k}}}(t)}{\sum _{{\varvec{k}}} [\mathcal M_{{\varvec{k}}}(t)+{\mathcal {M}}^*_{{\varvec{k}}}(t)]}, \end{aligned}$$

where conversion $\chi (t)$ is defined as

36 $$\begin{aligned} \chi (t)=1-\frac{{\mathcal {M}}_\mathbf{0}(t)}{{\mathcal {M}}_\mathbf{0}(0)}, \end{aligned}$$

See Table 1 for the numerical values used in the examples.

### Mathematical description: free-radical photopolymerisation

We consider the photopolymerisation of mono- and diacrylates and derive the differential equations for the concentration of monomer species at *N* discrete time intervals in a similar way as in the preceding section. The concentrations are used to construct the coloured degree distribution.

#### ODEs with colours for free-radical photopolymerisation

The reaction mechanism for polymer species can be found in Ref.^13^. We characterise the state of a monomer unit ${\mathcal {M}}_{v,r,{\varvec{k}}}$ by its number of vinyl groups *v*, radicals *r*, and the edge count vector ${\varvec{k}}=(k_1,\dots ,k_{2N})$ where *N* is the number of time intervals. The covalent bond formed during polymerisation is represented by a directed edge oriented from the initial radical site to the initial vinyl site. In the case of photopolymerisation of mono- and diacrylates, the elements of the count vector are restricted to $k_i\in \{0,1,2\}, \, i=1,\dots ,2N$, as every monomer unit can form at maximum two in-edges and two out-edges.

The reaction mechanism is summarised as follows:

- Photoinitiation of the initiator $I_2$ and formation of two initiator radicals *I* per initiator:
  $$\begin{aligned} I_2 \xrightarrow {k_\text {d}} 2I. \end{aligned}$$
- Initiation of a vinyl group by reaction with the initiator radical:
  $$\begin{aligned} I+ {\mathcal {M}}_{v,r,{\varvec{k}}} \xrightarrow {vk_\text {ini}} {\mathcal {M}}_{v-1, r+1,{\varvec{k}}}. \end{aligned}$$
- Propagation (including crosslinking) by the reaction of the radical with a vinyl group and formation of an edge of colour *c*:
  $$\begin{aligned}&{\mathcal {M}}_{v,r,{\varvec{k}}}+{\mathcal {M}}_{v',r',{\varvec{k}}'}\xrightarrow {k_\text {p}} \\&\,\,\,\,\,\,\,\,\,\,\,\mathcal M_{v,r-1,{\varvec{k}}+\Delta {\varvec{k}}_{0,1}(c)} + \mathcal M_{v',r',{\varvec{k}}'+\Delta {\varvec{k}}_{1,0}(c)}. \end{aligned}$$
- Termination by disproportionation by the reaction of two radicals:
  $$\begin{aligned}&{\mathcal {M}}_{v, r,{\varvec{k}}} + \mathcal M_{v',r',{\varvec{k}}'} \xrightarrow {rr'k_\text {td}} \mathcal M_{v, r-1,{\varvec{k}}} + {\mathcal {M}}_{v', r' -1,{\varvec{k}}'}. \end{aligned}$$

The corresponding system of ODEs reads as:

37 $$\begin{aligned} \begin{pmatrix} \dot{{\mathcal {M}}}_{v,r,{\varvec{k}}}(t)\\ {\dot{I}}_2(t)\\ {\dot{I}}(t)\\ \end{pmatrix} = \begin{pmatrix} G_{v,r,{\varvec{k}}}(t)\\ -k_\text {d} I_2(t), \\ 2k_\text {d} I_2(t)-k_\text {i} I(t) c_v(t)\\ \end{pmatrix}, \end{aligned}$$

where

38 $$\begin{aligned} G_{v,r,{\varvec{k}}}(t)&:= k_\text {ini}I(t)\big [ (v+1) {\mathcal {M}}_{v+1,r-1,{\varvec{k}}}(t) - v {\mathcal {M}}_{v,r,{\varvec{k}}}(t) \big ]\\&\quad +k_\text {p}c_r(t)\big [(v+1) {\mathcal {M}}_{v+1,r-1,{\varvec{k}}-\Delta {\varvec{k}}_{1,0}(c)}(t) \\&\quad - v {\mathcal {M}}_{v,r,{\varvec{k}}}(t) \big ] \\&\quad + k_\text {p}c_v(t)\big [(r+1) {\mathcal {M}}_{v,r+1,{\varvec{k}}-\Delta {\varvec{k}}_{0,1}(c)}(t) \\&\quad - r {\mathcal {M}}_{v,r,{\varvec{k}}}(t) \big ] \\&\quad +2k_\text {td}c_r(t)\big [(r+1) {\mathcal {M}}_{v,r+1,{\varvec{k}}}(t) - r {\mathcal {M}}_{v,r,{\varvec{k}}}(t) \big ]. \end{aligned}$$

The radical concentration $c_r(t)$ and vinyl concentration $c_v(t)$ in the system are given by

39 $$\begin{aligned}&c_r(t)=\sum _{v,r,{\varvec{k}}} r{\mathcal {M}}_{v,r,{\varvec{k}}}(t),\\&c_v(t)=\sum _{v,r,{\varvec{k}}} v\mathcal M_{v,r,{\varvec{k}}}(t). \end{aligned}$$

The polymerisation progress is quantified by the vinyl conversion

40 $$\begin{aligned} \chi (t)=1-\frac{c_v(t)}{c_v(0)}, \end{aligned}$$

with $c_v(0)$ the initial vinyl concentration. The remaining initial concentrations are given as $I_2(0)$, *I*(0), and $\mathcal M_{v,r,{\varvec{k}}}(0)=0$ for all $v,\,r,\,{\varvec{k}}$ except for ${\mathcal {M}}_{2,0,{\varvec{k}}_{0,0}}(0)=c_\text {di}\mathcal M_0$ and $\mathcal M_{1,0,{\varvec{k}}_{0,0}}(0)=c_\text {mono}{\mathcal {M}}_0$. The fractions $c_\text {di}$ and $c_\text {mono}$ refer to the fractions of di- and monovinyls in the systems. All numerical values are presented in Table 1.

After solving the system of ODEs, the degree distribution is computed:

41 $$\begin{aligned} u({\varvec{k}})=\frac{\sum _{v,r}\mathcal M_{v,r,{\varvec{k}}}(t)}{\sum _{v,r,{\varvec{k}}}\mathcal M_{v,r,{\varvec{k}}}(t)}. \end{aligned}$$

#### Special case: cross-linked living polymerisation

Living polymerisation is a simplified case of the free-radical polymerisation: As living polymerisation does not include initiation and termination reaction, the respective rate constants are set to zero, $k_\text {ini}=0, \,k_\text {td}=0$. Also, initiator and initiator radicals are not present in the system, hence $I_2(0)=0$, $I(0)=0$. After applying these changes, the system of ODEs (37) simplifies to

42 $$\begin{aligned} \dot{{\mathcal {M}}}_{v,r,{\varvec{k}}}(t)&= k_\text {p} c_r(t) \big ((v+1) {\mathcal {M}}_{v+1,r-1,{\varvec{k}}-\Delta {\varvec{k}}_{1,0}(c)}(t) \\&\quad - v {\mathcal {M}}_{v,r,{\varvec{k}}}(t) \big ) \\&\quad + k_\text {p} c_v(t) \big ((r+1) {\mathcal {M}}_{v,r+1,{\varvec{k}}-\Delta {\varvec{k}}_{0,1}(c)}(t) \\&\quad - r {\mathcal {M}}_{v,r,{\varvec{k}}}(t) \big ). \end{aligned}$$

The initial conditions are given by

- $${\mathcal {M}}_{2,0,{\varvec{k}}_{0,0}}(0)=c_\text {di}(1-R_0)^2{\mathcal {M}}_0$$,
- $${\mathcal {M}}_{1,1,{\varvec{k}}_{0,0}}(0)=c_\text {di}2R_0(1-R_0)){\mathcal {M}}_0$$,
- $${\mathcal {M}}_{0,2,{\varvec{k}}_{0,0}}(0)=c_\text {di}R_0^2{\mathcal {M}}_0$$,
- $${\mathcal {M}}_{1,0,{\varvec{k}}_{0,0}}(0)=c_\text {mono}(1-R_0){\mathcal {M}}_0$$,
- $${\mathcal {M}}_{0,1,{\varvec{k}}_{0,0}}(0)=c_\text {mono}R_0{\mathcal {M}}_0$$,
- $${\mathcal {M}}_{v,r,{\varvec{k}}}(0)=0$$ elsewhere,

with $c_\text {di}$ and $c_\text {mono}$ the fraction of di- and monofunctional monomers and $c_\text {di}+c_\text {mono}=1$.

### Randomised matrix $\tilde{{\varvec{M}}}$

Matrix $\tilde{{\varvec{M}}}$ is defined in a similar manner as ${\varvec{M}}$ in Eq. (4),

43 $$\begin{aligned} {\tilde{M}}_{i,j}=\frac{{\tilde{\mu }}_{i,j}}{{\tilde{\mu }}_{j}}-\delta _{i,j},\;i,j=1,\dots ,2N \end{aligned}$$

with ${\tilde{\mu }}_{i,j}$ defining the second mixed moments and ${\tilde{\mu }}_{j}$ the first moments of a 2*N*-variate degree distribution with randomised colours. The randomised degree distribution is based on the uncoloured directed degree distribution $u(k_1,k_2)$ with every half-edge having equal probability of having any colour *i* with $i=1,\dots ,N$, which results in a multinomial distribution.

In order to mathematically describe the randomised system, we define the uncoloured directed degree distribution as the distribution that is obtained from an originally coloured degree distribution by neglecting the colour, but retaining the orientation:

44 $$\begin{aligned} u(x,y)=\sum _{\Omega _{x,y}} u({\varvec{k}}), \end{aligned}$$

where the summation is performed over

$$\begin{aligned} \Omega _{x,y}=\{\mathbf{k} |\sum _{i>0} k_{2(i-1)}=x, \sum _{j>0} k_{2j}=y \} \end{aligned}$$

containing all configurations of vector $\mathbf{k}$ with *x* in-edges and *y* out-edges regardless their colour.

The first moments of the randomised degree distribution are given by

45 $$\begin{aligned} {\tilde{\mu }}_{2(i-1)}&=\sum _{k_1,k_2}\sum _{k_1'=0}^{k_1} k_1' u(k_1,k_2) \\&\times \left( {\begin{array}{c}k_1\\ k_1'\end{array}}\right) \left( \frac{1}{N}\right) ^{k_1'} \left( \frac{N-1}{N}\right) ^{k_1-k_1'}, \end{aligned}$$

46 $$\begin{aligned} {\tilde{\mu }}_{2i}&=\sum _{k_1,k_2}\sum _{k_2'=0}^{k_2} k_2' u(k_1,k_2) \\&\times \left( {\begin{array}{c}k_2\\ k_2'\end{array}}\right) \left( \frac{1}{N}\right) ^{k_2'} \left( \frac{N-1}{N}\right) ^{k_2-k_2'}. \end{aligned}$$

In order to characterise the second mixed moments, we distinguish between the second moments with same half-edge types,

47 $$\begin{aligned} {\tilde{\mu }}_{2(i-1),2(i-1)}=&\sum _{k_1,k_2}\sum _{k_1'=0}^{k_1} k_1'^2 u(k_1,k_2) \\&\times \left( {\begin{array}{c}k_1\\ k_1'\end{array}}\right) \left( \frac{1}{N}\right) ^{k_1'} \left( \frac{N-1}{N}\right) ^{k_1-k_1'}, \end{aligned}$$

48 $$\begin{aligned} {\tilde{\mu }}_{2i,2i}=&\sum _{k_1,k_2}\sum _{k_2'=0}^{k_2} k_2'^2 u(k_1,k_2) \\&\times \left( {\begin{array}{c}k_2\\ k_2'\end{array}}\right) \left( \frac{1}{N}\right) ^{k_2'} \left( \frac{N-1}{N}\right) ^{k_2-k_2'}, \end{aligned}$$

the second moments with same half-edge orientation,

49 $$\begin{aligned} {\tilde{\mu }}_{2(i-1),2(j-1)}=&\sum _{k_1,k_2}\sum _{k_1'=0}^{k_1} \sum _{k_1''=0}^{k_1'} k_1''(k_1'-k_1'') u(k_1,k_2) \\&\times \left( {\begin{array}{c}k_1\\ k_1'\end{array}}\right) \left( \frac{1}{N}\right) ^{k_1'} \left( \frac{N-1}{N}\right) ^{k_1-k_1'}, \end{aligned}$$

50 $$\begin{aligned} {\tilde{\mu }}_{2i,2j}=&\sum _{k_1,k_2}\sum _{k_2'=0}^{k_2} \sum _{k_2''=0}^{k_2'} k_2''(k_2'-k_2'') u(k_1,k_2) \\&\times \left( {\begin{array}{c}k_2\\ k_2'\end{array}}\right) \left( \frac{1}{N}\right) ^{k_2'} \left( \frac{N-1}{N}\right) ^{k_2-k_2'}, \end{aligned}$$

and the second moments with different types and orientation,

51 $$\begin{aligned} {\tilde{\mu }}_{2(i-1),2j}=&\sum _{k_1,k_2}\sum _{k_1'=0}^{k_1}\sum _{k_2'=0}^{k_2} k_1'k_2' u(k_1,k_2) \\&\times \left( {\begin{array}{c}k_1\\ k_1'\end{array}}\right) \left( \frac{1}{N}\right) ^{k_1'} \left( \frac{N-1}{N}\right) ^{k_1-k_1'}\\&\times \left( {\begin{array}{c}k_2\\ k_2'\end{array}}\right) \left( \frac{1}{N}\right) ^{k_2'} \left( \frac{N-1}{N}\right) ^{k_2-k_2'}, \end{aligned}$$

with $${\tilde{\mu }}_{2(i-1),2j}={\tilde{\mu }}_{2j,2(i-1)}$$.
